# Indents

**Published:** 1905-11-15

**Authors:** 


### INDENTS.

☞ **Brief Articles of Technical or Scientific Value will receive notice and comment. Contributions invited.**

**Quick Investment Material.** For quick investment for soldering bands to clasp the teeth, the following mixture may be kept on hand ready prepared: Equal parts of prepared chalk and fine sand, kneaded in glycerin, making a plastic mass similar to mouldine.—*Hints.*

**Fatalities.** July 10th, a prominent young physician of Pittsburg, died from heart failure in a dentist's chair after the administration of chloroform, but before the extraction was performed.—July 7th, a middle-aged man suffered a stroke of paralysis at Wichita, Kan., while having a tooth extracted and died a few hours later.—July 25th, a young woman submitted to an operation for a cleft palate at a clinic before the Lewis and Clark Dental Congress, and died without recovering from the anesthetic —July 25th, a woman, forty-nine years old, had sixteen teeth extracted by a Syracuse, N. Y., dentist, chloroform being the anesthetic used, and died without recovering from the drug.—August 4th, a young woman, twenty-two years old, died from nervous collapse brought on by a severe tooth-ache. The day previous a dentist tried to relieve her condition, but she was so nervous that he could do nothing.—August 2, a child, ten years old, died at Philadelphia from blood poisoning, resulting from an abscess on the root of a tooth.—July 24, a man, forty years old, died in Chicago from blood poisoning, which is alledged to have been caused by the careless work of a dentist.—July 14, a young man at Jeffersonville, Ind., died from hemorrhage following the extraction of a tooth.—July 3, a man died at Cincinnati from a peculiar cause. Two months previous, while eating, he broke off the crown of a tooth which was badly decayed. No attention was paid to it and a little while later an abscess developed. He paid no attention to this until it became very painful, and by that time the glands of his neck were involved and his whole system was so poisoned that he died in the hospital.—*Digest.*

**Less Advice, More Cash.** There is a tendency on the part of the profession generally to criticise the educational institutions unjustly. The following remarks by that veteran dentist and scholar, Dr. Wm. H. Truman, are timely and to the point. Few schools of the various callings which devote themselves to higher education are dependent for maintenance on the students' fees to the same extent as are the dental colleges. The education of a dentist is more expensive than most other callings, because of the combination of technical and scientific requirements. Dental students require larger quarters, more expensive equipment and a higher grade of teachers than ordinary professional training schools. It requires lots of money to equip and maintain an ideal dental school, and our colleges could do much more effective teaching if they were not financially limited. Modern teaching requires much expensive apparatus and fewer students in the classes which means more expensive instructors. The "lecturer" is now giving way to the "demonstrator" and training takes the place of dialectics. Our professors are not financially compensated and many instructors serve for nothing, except the name of a teaching connection, which they invariably sever as soon as their practice requires all their time. With sufficient funds our schools would be able to develop and retain teachers of promise and recognized ability, who would serve the college as well as the profession and public more acceptably.

If our profession really wants better schools, they can be promoted by the necessary financial support. Our profession has made enormous advances in practice, but the methods of imparting instruction thoroughly have been given scant attention. Of the many rich dentists who have died, very little accumulated wealth has been given by them toward fostering dental education. I know of only two bequests to dental education and these were less than $1,000. Our dental schools have proved their usefulness and worthiness; they have done well but can and will do better if furnished more money and less advice.—August *Cosmos*.

**Cement and Broken Porcelains.**

In this connection, although it is foreign to the subject to an extent, I wish to speak of what can be accomplished by the admixture of the materials other than the precipitated metals. Those metallic mixtures do not have a satisfactory color for the anterior teeth; so while we can use them in the posterior teeth, for the anterior we must look for something else. Here again the nature of the crystal or quality of material comes into play. I have secured a great deal of satisfaction for a few years past, especially quite recently from the admixture of such materials as properly precipitated alumina or porcelain powders for inlay and other work.

From the fact that we can obtain porcelain powders in various colors, we have in that the material which we can use to the greatest advantage for these processes, and I have found after over four years' observation of fillings in which a large amount of pulverized porcelain was incorporated with zinc oxyphosphate, that results have been brought about which were out of proportion to what might have been expected with plain oxyphosphate. I will say that it has been my experience with cement that on the occlusal surface of a molar or bicuspid it will last several times as long as upon an approximal surface of an incisor. In those cavities the destruction of the filling is brought about from the stagnation of fluids and the fermentation and putrefaction of food, but where such fillings of porcelain and oxyphosphate were put in more than four years ago, the results have been very satisfactory. I find I have made fillings which to-day are practically perfect, which, had they been made of plain oxyphosphate, would have needed renewal some time since. The theory, as near as I can advance it, is that the porcelain particles—after we have used about equal bulks of porcelain body and cement powder—are so near together as to make something like a very fine inlay joint, and the cement does not seem to waste to the depth of one layer of porcelain particles, so that in a short time we have a surface which is apparently all porcelain, and resembling an underbaked porcelain inlay. I myself have been deceived, having forgotten making certain fillings, and when some of these cases came back I would go to my book to see what I had done, finding that I had made one of these fillings I have mentioned. If it is as I say, that the porcelain particles are so close together as to form a very fine joint, we have a very valuable method in certain cases where we have thin walls which can be retained in that way. I have applied it in just such cases where gold had failed, and on the removal of fillings after secondary caries, I had such a condition that the walls were transparent, and operating in the way I have mentioned I have seen extremely satisfactory results in every way.—W. V. B. Ames, in *Cosmos*.

**Pointers on Cocain. Anesthesia for the Extirpation of Pulps.**

In my experience there have been cases in which it was impossible to obtain anesthesia with cocain. I believe the explanation of that is to be found in an hyperemic condition of the pulp. The pulp is an extremely hyperemic condition, under unusual blood pressure, the bloodvessels filled, and crowding the tissues; it will be impossible to make that tissue take up cocain. I have worked and worked over them. In some cases I have succeeded in extirpating the pulp by main force and brutality, and in other I have applied arsenic and removed the pulp without trouble. As the result of my experience I have come to know that cocain is useless, if the symptoms indicate a chronic, long-continued inflammation, or an extremely acute hyperemia. In such cases I employ either a treatment calculated to reduce the hyperemia, or an application of arsenic, depending upon the symptoms which precede it. If the patient has been suffering acute pain for twenty-four hours previously, to make an arsenic application at that sitting would result in a worse toothache than ever for the next twenty-four hours. But if it is a relapsing pain, appearing and disappearing, an arsenic application without pressure will not cause unbearable pain. If the symptoms indicate a condition which is certain to result in pain from the arsenic application, I use 95 percent carbolic acid, or beechwood creosote, or after twenty-four hours remove it, and try the cocain again. Often the cocain will work at the second sitting where it failed at the first. If it does not, the arsenic almost invariably will. I have seen one or two instances of trouble following the removal of pulps with cocain and immediate root-filling. One was shown me about two years ago. It was a lower third molar, from which the operator, a very successful man, had removed the pulp under cocain anesthesia, and filled the root at the same sitting. His description of the condition for the forty-eight hours following was graphic. At the end of that time he extracted the tooth, and he showed it to me with gutta-percha protruding from all the roots. My explanation of that was, that in the application of the cocain he had anesthetized not only the pulp but the pericementum, and consequently failed to obtain his signal for complete root filling.—that is, the symptom of slight pain which he had learned to depend upon as indicating that his root-filling had reached the apex, and so he had pushed the root filling through the apex. I believe, on the other hand, there is pain after root filling, because of imperfect removal of the pulp, leaving portions in the tissue, either alongside the root filling or beyond it. I think you will always have trouble from both these sources, no matter how skillful the operator may be, because root canals are not always straight. —F. B. Noyes, *Dental Review*.

**The Lewis and Clark Dental Congress.** Dental congresses being designed largely to act as educators of the profession and the public, it may not be out of place to mention a few of the lessons which it seemed to us were taught by the Lewis and Clark Dental Congress.

The success of the meeting, from every standpoint, professional, social and financial, leads us to recognize, how even as great an undertaking as this, may be brought successfully through its various stages of development and life, by the steady, untiring, unselfish effort of a few determined men. Apparently no one "did politics" or accused any one else of "doing" them; there was no scandal, no newspaper notoriety, and nothing that smacked of charlatanism, to mar the clean, pleasant record of the meeting.

Those who had worked so hard to insure the success of the meeting were appropriately honored by the election to office, and no one objected. Harmony seemed to be the guiding spirit of the meeting, and no doubt contributed largely to its success.

The exhibits were an object lesson, or rather many lessons, in all that pertains to equipment and office supplies, and contributed in a large degree to the pleasure and profit of the members. We trust this profit may not be all on their side however, but that those who spent so much time and money in exhibits and exhibitor's clinics, may reap a just and fair reward.

The clinics, as in all recent meetings, illustrated how great a part electricity plays in office equipment and operative and prosthetic procedure, and all those in practice must realize that this subtle force is one of the powers that are steadily moving dentistry onward. The attention given to inlays, porcelain and gold, shows how large an amount of studious effort is being expended to make the operation of "filling teeth" easier and better; easier, for both patient and operator, and better for all parties concerned, including the teeth.

It seems to us that the gold inlay, as exhibited and demonstrated by gentlemen from the East, will shortly do away with that horror of the dental chair, the two or three hour gold filling, and the hardly lesser horror of large, unsightly, amalgam fillings. The literary portion of the program was, as will be evident to those who read the published proceedings, above the average in scholarship, and evidence of scientific research. We feel that it marks a decided advance in the educational standing of the dental profession and already shows evidence that the effort for better, broader, more efficient professional culture, is bearing fruit. To refer to all the lessons of the Congress would necessitate the publication in our editorial columns of the entire transactions, but those we have mentioned were prominent features and above all else the meeting was a pronounced success. We are glad to have been in attendance.—Editorial in *Pacific Gazette.*

**New Interdental Splint.**

The splint devised by Russ is made of spring brass, three sixty-fourths of an inch in thickness. Each clamp consists of two parts, a chin-piece and a piece bent at right angles, making a long and short arm. The chin-piece is wider at one end to prevent its slipping when incorporated into the plaster of Paris dressing. It is eight cm. long and provided with a number of holes so that the plaster of Paris may sink into them, thus making the dressing more secure. The right angled piece is one cm. wide, while the arms measure seven and a half cm. and five cm. respectively. The end of the short arm is provided with a simple lock which fits into a corresponding lock in the chin-piece. Three or four holes should be bored into the long arm. When these two parts are locked they form a clamp which permits of easy adjustment. This adjustment is accomplished by means of a round head brass machine-screw six cm. in length and carrying a winged nut. This fits into holes in the long arm and the chin-piece at one and one-half cm. distance from the short arm. After the clamp has been made, it should be heavily nickel plated. The clamp has been found to be applicable to thirty-five out of fifty adult jaws. For the other cases, a splint with a short arm five and one-half cm. answers. The splint is applied with the patient lying in the dorsal position on the operating table. The face is shaven clean and the fractures reduced. A thin layer of felt or layers of sheet wadding are then fitted neatly over the chin and under the jaw, extending as far back as the rami. Over this the plaster of Paris bandage is carefully folded, the operator rubbing the plaster well as he proceeds. The bandage is carried back on each side to the ramus, where the turns are held by an assistant or by a patient. When the jaw is well covered the splints are applied, the parts being held together by the screws and nuts. The long arms are placed directly over the molar and bicuspid teeth, extending as far back as the last molars and emerging at the corners of the mouth. The chin-pieces are pressed into the plaster. They should run along the body of the jaw. The apparatus being in position, a few more turns of the bandage are taken and the dressing completed by rubbing in dry plaster of Paris. Accurate coaptation is desired. When dry, the chin-pieces are detached from the interdental portions and the plaster of Paris dressing removed and trimmed to the desired shape. The dressing is then reapplied to the jaw. The interdental portions of the splints, together with some dental compound, such as is commonly used by dentists in taking impressions, are thrown into hot water. When sufficiently softened, the dental compound is applied along the under surface of the interdental arms, and these are quickly placed on the teeth, in the same position as in the first application, and pressed firmly down so that the splints rest on an even bed, the dental compound filling up the holes which have been bored into the long arms. The short arms are then locked to the chin-pieces, the screws re-applied, and the apparatus tightened by means of the winged nuts.—R. Russ, in August *Annals of Surgery.*

**The Prosthodontist.** The specialist in orthodontia has made a special study in his art, and his aim is to re-arrange the natural dental organs so as to establish occlusion, a pleasing alignment, and, as nearly as possible, harmony of the features. The prosthodontist will be a man proud of his calling, and he will do all that the orthodontist does, except that his restorations must be with the aid of artificial substitutes. He will establish occlusion, a pleasing alignment and a matching of the artificial with the natural, and, withal, he will have the artistic temperament which will demand that his work shall restore harmony of the features.

Let us ask the question, "What is the desire of the patient who applies for artificial teeth?" Primarily, he wishes teeth. Let that be borne in mind. Too many men—men of skill and ability—forget this. In solving the problem presented, too often the mechanical genius devotes all his energies to the structure which is to carry the teeth. Shall it be plate or bridge. If the latter, shall it be fixed or removable? If removable, of what nature shall the clasps be? These queries decided he goes to work, and perhaps he produces a fixture which is the acme of his art—of the mechanical art of dentistry. The final step, the choice and assembly of the teeth, he treats as a simple proposition. There is an attempt at matching color, but a shade one way or the other matters little. The length and width of each tooth receives some thought; the form of it, little, if any. The occlusion is all too often a useless antagonism, all morsal surfaces being ground to shapeless smoothness. The result may be a wonderfully adapted mechanical substitute for the lost organs; one that fits, and is firmly attached; perhaps even of some use in mastication. But, oh! How often, after all, is it the product of the mechanical dentist, and not of the prosthodontist? How readily the friends of the patient detect the falsity of this appliance, of which no doubt the dentist was so proud!

Two occurences recently, coming close together, have suggested the theme of this editorial. First, a visit from the patient of another dentist. A gentleman highly esteemed and a man of great ability. The case at the outset must have been most difficult. Special porcelain crowns had been made, so shaped as to form ingenius abutments, about which a removable fixture was adapted. All was accurately and beautifully achieved. But the teeth? The baby grand-children will know them to be false. Wrong in size, wrong in shape, wrong in color, the dentist yet further decorated them with little simulations of tobacco stains. Oh, the poverty of such art, and the uselessness of it! Think a moment; why should an almost complete set of teeth be stained with tobacco? To advertise the fact that the man had this unclean habit?

The other visit was from the agent of a mechanical laboratory, who presented specimens of his work. He little appreciated that these specimens would prevent any dentist of artistic temperament from sending work to such an establishment. Nice straight teeth, in nice straight rows. Full sets articulated so as to compel an up and down movement in mastication. But the finish, ah! the finish! All gold surfaces shown like mirrors. All rubber attachments polished to the most inaccessible corners. A factory product, but then what is a mechanical laboratory but a factory? A factory for false teeth, forsooth!

Kind genius of dentistry hasten the day of departure of the mechanical dentist, and the man ashamed of making false teeth; so ashamed that he must have the major part of the work, for which he takes a fee, done in a factory. Hasten the coming of the prosthodontist, the artist in the production of artificial teeth, proud of his calling, and earning with his own hand and brain the money of his client.—Editorial in *The Items.*

